# Application of PTFE/Al Reactive Materials for Double-Layered Liner Shaped Charge

**DOI:** 10.3390/ma12172768

**Published:** 2019-08-28

**Authors:** Haifu Wang, Huanguo Guo, Baoqun Geng, Qingbo Yu, Yuanfeng Zheng

**Affiliations:** State Key Laboratory of Explosion Science and Technology, Beijing Institute of Technology, Beijing 100081, China

**Keywords:** shaped charge, reactive materials, PTFE/Al, penetration behavior, thick steel target

## Abstract

The penetration enhancement behaviors of a reactive material double-layered liner (RM-DLL) shaped charge against thick steel targets are investigated. The RM-DLL comprises an inner copper liner, coupled with an outer PTFE (polytetrafluoroethylene)/Al reactive material liner, fabricated via a cold pressing/sintering process. This RM-DLL shaped charge presents a novel defeat mechanism that incorporates the penetration capability of a precursor copper jet and the chemical energy release of a follow-thru reactive material penetrator. Experimental results showed that, compared with the single reactive liner shaped charge jet, a deeper penetration depth was produced by the reactive material-copper jet, whereas the penetration performance and reactive material mass entering the penetrated target strongly depended on the reactive liner thickness and standoff. To further illustrate the penetration enhancement mechanism, numerical simulations based on AUTODYN-2D code were conducted. Numerical results indicated that, with increasing reactive liner thickness, the initiation delay time of the reactive materials increased significantly, which caused the penetration depth and the follow-thru reactive material mass to increase for a given standoff. This new RM-DLL shaped charge configuration provides an extremely efficient method to enhance the penetration damage to various potential targets, such as armored fighting vehicles, naval vessels, and concrete targets.

## 1. Introduction

In general, PTFE-based reactive materials are formed by mixing active metal powders within the fluoropolymer binder and then are consolidated via a pressing/sintering process, such as PTFE/Al [1,2], PTFE/Ti [3], and PTFE/Cu [4]. These reactive materials are a class of energetic materials that are formulated to release their chemical energy under intense dynamic loadings or high pressures and high strain rates [5,6]. Recently, studies on these reactive materials have mainly examined the energy release characteristics [7,8], the impact initiation behaviors [9,10], and the microstructural and mechanical performance [11,12]. In particular, the penetration performance and enhanced damage effects of reactive material projectiles impacting aluminum plates [13,14,15], covered explosives [16], and fuel-filled tanks [17], as well as the interval rupturing damage effects of reactive-materials filled projectiles to multi-spaced aluminum plates [18]. 

Additionally, shaped charges with reactive material liners are a novel application of reactive materials; they can form a high-velocity jet or an explosively formed projectile (EFP) to achieve enhanced structural damage to concrete and steel targets, or greater behind-armor effects [19,20]. Compared with the traditional metal jet, which penetrates the target using only kinetic energy, the reactive material jet first penetrates the target with its kinetic energy and then releases its chemical energy when inside of the target, thus producing extremely large amounts of damage to the desired targets. Owing to the unique and excellent performance, shaped charges with reactive material liners have been studied extensively over the past twenty years. Baker and Daniels investigated the reactive jet formation behavior by using an X-ray pulse and discussed the influence of the explosive type and shape on jet formation properties; they also experimentally analyzed the effects of standoff on penetration performance [21]. EFP with reactive materials, which can achieve enhanced behind-armor effects, was analyzed by experiments and numerical simulations [20]. Further studies on reactive liner shaped charges indicated that the reactive material mass entering the penetration hole and the initiation location of the reactive jet significantly influenced the overpressure inside the targets or the damage mechanism of concrete and steel targets [22,23,24]. In addition to PTFE/Al reactive materials, the jet formation and penetration behaviors of PTFE and PTFE/Cu reactive liner shaped charges have also been researched by experiments and simulations [4,25,26].

However, although this class of reactive material jet can form a larger hole-diameter on the target, and its deflagration reaction inside of the target will produce enhanced structural damage, its penetration depth is always lower, which makes it difficult for a reactive liner shaped charge to efficiently penetrate a thicker steel target [27,28]. This is mainly because the chemical reaction of the reactive materials occurs before the jet perforates the thick steel casing of targets, resulting in the reactive materials not entering the target and not producing enhanced behind-armor effects. In contrast, the traditional inert metal jet (such as a copper jet, tantalum jet, or tungsten jet) can produce deeper penetration, yet the subsequent residual jet cannot effectively damage weaponry when inside of the target, as it uses only kinetic energy. Therefore, enhancing the behind-armor damage to a thicker target by incorporating the penetration capability of a metal jet and the chemical energy release of reactive materials is an extremely urgent and important engineering problem.

Compared with the traditional single-layered liner shaped charge, the energy conversion and absorption mechanism of a double-layered liner shaped charge, as well as its utilization of explosive chemical energy, is more reasonable and sufficient, thus improving penetration performance [29,30,31,32]. Hence, based on a traditional single-layered reactive liner shaped charge, we added a copper liner to the inner side of a reactive material liner to form a novel reactive material-copper liner (RM-CL) shaped charge. Presently, research on the jet formation of this double-layered liner shaped charge and its penetration enhancement damage for thick steel targets is scarce. In particular, the influence of the reactive materials’ chemical response on the penetration performance and damage mechanism is not well understood.

This paper presents such research, beginning with a description of the penetration process of the RM-CL shaped charge impacting thick steel targets. Subsequently, a series of experiments are conducted to investigate its penetration performance and damage effects on the targets. Finally, the influence of reactive liner thickness on the initiation delay time of the reactive materials was discussed, and the initiation delay time affecting the penetration performance and the damage enhancement mechanism were analyzed further.

## 2. Description of Penetration Behavior

**Figure 1:**
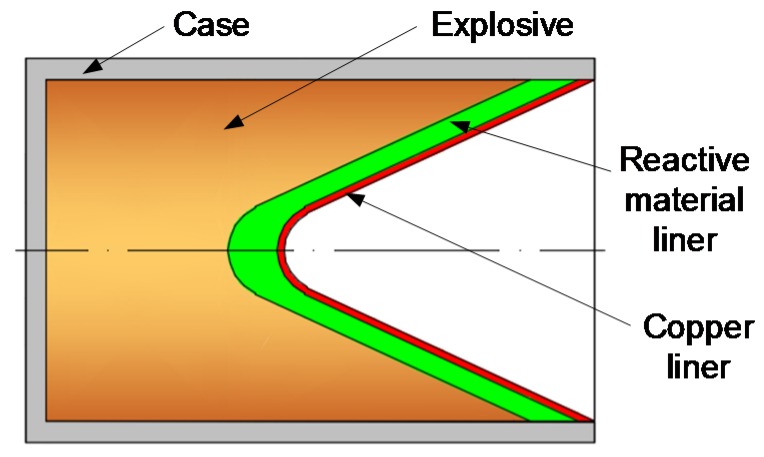
Typical configuration of a reactive material-copper liner (RM-CL) shaped charge.

**Figure 2:**
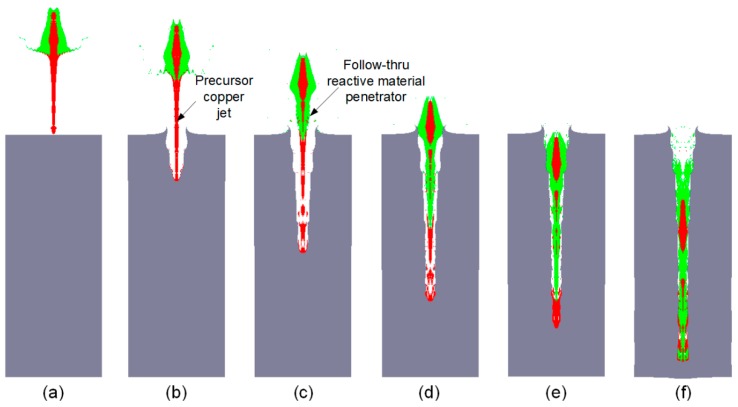
Typical penetration process of a RM-CL shaped charge impacting steel target: (**a**) Reactive material-copper jet (RM-CJ) formation characteristic before impacting; (**b**) precursor copper jet begins to penetrate the target; (**c**) and (**d**) show the head and slug of the reactive material penetrator starting to enter the penetration hole, respectively; (**e**) copper jet piles up and all reactive materials enter the target; and (**f**) shows the penetration at the termination stage.

The RM-DLL shaped charge is comprised of three basic parts: The case, the explosive, and a double-layered liner. This double-layered liner consists of two elements: An outer liner made of reactive materials and an inner liner (away from the explosive) made of high-density metal materials. The penetration enhancement behaviors of the RM-DLL shaped charge against a steel target are significantly influenced by the action condition of the projectile and target; specifically, the thickness ratios of the reactive material liner to the metal liner, the material of high-density metal liner, and the standoff. However, different conditions always involve the same penetration process. Therefore, this phenomenon can be explained using one example for all conditions. This simulated condition is that the double-layered liner is composed of an outer reactive material liner and an inner copper liner, with corresponding wall thicknesses of 4 mm and 1 mm, respectively, as shown in Figure 1. The RM-CL shaped charge impacts a 250-mm-thick steel target when the standoff is 2.0 CD (charge diameter). Figure 2 shows the simulated results based on AUTODYN-2D code.

The penetration enhancement behavior of the RM-CL shaped charge against the steel target is very complex, and it incorporates the damage mechanisms of both kinetic energy and chemical energy to achieve an extremely efficient damage technology for thick steel targets. This enhanced damage process can be divided into three main stages: The jet formation, the jet penetrating target, and the reactive materials releasing their chemical energy during or at the termination of the penetration process.

During the jet formation stage, under the detonation pressure of the main charge, the outer reactive material liner first compresses the inner copper liner, resulting in the copper liner mainly forming a high-velocity precursor jet. In this stage, the high-density metal materials of the inner liner and the thickness of the reactive liner that is used are irrelevant, as the reactive materials do not enter the precursor jet but become a major part of the slug, as seen in Figure 2a. At the stage of open cratering and quasi-static penetrating, it is the precursor copper jet that mainly penetrates the steel target, as illustrated in Figure 2b. As the precursor jet continues to penetrate the target, the reactive material penetrator follows the copper jet into the penetration hole, as shown in Figure 2c,d. Figure 2e indicates that, at this time, all reactive materials enter the penetrated target, and the copper jet is basically consumed and piled up at the bottom of the penetration hole. As the penetration time progresses, the reactive material penetrator begins to impact the target until the termination of the penetration, as depicted in Figure 2f. Thus, according to the numerical simulations, the reactive material penetrator has a negligible effect on the penetration performance, whereas the entrance hole-diameter and penetration depth are predominantly determined by the precursor copper jet.

However, under the high-pressure of the explosive detonation, the temperature inside the reactive materials rises during the compressed and closed process of the liner, which can activate the high-polymer PTFE molecules to decompose and release C_2_F_4_. As time passes, the metal powder Al will experience a redox reaction with the fluoride, releasing a large number of reactive heat and gas products. Under the oxygen deficient system, the main reactions include:

[–C_2_F_4_–] n → n C_2_F_4_ (g) (1)

4Al + 3C_2_F_4_ → 4AlF_3_ (g) + 6C (2)

Based on the platform of REAL/ASTD, for the 73.5 wt.% PTFE/26.5 wt.% Al composite, PTFE and Al will mainly react to form AlF_3_ and C. The reactions between Al and C_2_F_4_ release large amounts of chemical energy, and the theoretical value of the reactive heat is approximately 5949 kJ/kg, whereas that for the amount of deflagration gas products is approximately 3.84 L/g. It should be noted that the process will take some time to be achieved, including the temperature rise of the reactive liner, the PTFE decomposition, and the final violent deflagration reaction; the period of time from activation to violent initiation is called the initiation delay time of the reactive materials (*τ*). 

Based on the above description, one of the following situations will occur to cause the penetration termination. One situation is that, if the initiation delay time is less than the maximum penetration time (the time corresponding to Figure 2f, the reactive materials experience a deflagration reaction during the jet formation or penetration process. The other is that, after the termination of the penetration process, the reactive materials will experience a deflagration reaction. If the first situation leads to the penetration termination; the initiation delay time of the reactive materials is the primary determinant for the terminal damage performance of the RM-CL shaped charge. If the second case causes the penetration termination, all the reactive materials have entered the penetrated target, so the copper jet characteristics, such as the jet tip-velocity, will be critical to the penetration performance.

Thus, the RM-DLL shaped charge provides a novel defeat mechanism to enhance the terminal damage performance. For the traditional double-layered metal liner shaped charge used against armored fighting vehicles, the metal jet must perforate the thick steel casing with sufficient velocity, while the follow-thru jet must still achieve substantial residual velocity to guarantee effective damage inside of the target. In contrast, the RM-DLL shaped charge requires only the precursor metal jet to perforate the target casing and convey the follow-thru reactive material penetrator to enter the penetrated target, in which the reactive materials release large amounts of chemical energy to produce greater behind-armor effects. In addition, the charge caliber of the RM-DLL shaped charge can be engineered to decrease its size, which reduces the amount of explosive that must be carried by the warhead. Thus, the terminal damage performance of the RM-DLL shaped charge is not only strongly dependent on the precursor jet penetration capability but also on the blast effects of reactive materials, and the latter are dramatically affected by follow-thru reactive-material mass.

Furthermore, Figure 2 indicates that the reactive-material mass entering the penetrated target is primarily determined by the initiation delay time (*τ*). If *τ* is less than the jet formation time, although the RM-CL shaped charge can form a jet, the chemical reaction will occur before penetrating the target, which makes the penetration depth and follow-thru reactive-material mass decrease to zero (see Figure 2a). If *τ* is relatively smaller, the jet can produce a deeper penetration depth, but the reactive materials will still not enter the penetration hole; thus, the deflagration reaction will not enhance the damage to the target effectively (see Figure 2b,c). As time (*τ*) progresses, the follow-thru reactive-material mass gradually increases (see Figure 2d,e). Nevertheless, if the initiation delay time is sufficiently long, i.e., *τ* is larger than the maximum penetration time, the combined damage effects of kinetic and chemical energy can be maximized. 

## 3. Experiments of Penetration

### 3.1. Reactive Material-Copper Liner Specimens

**Figure 3:**
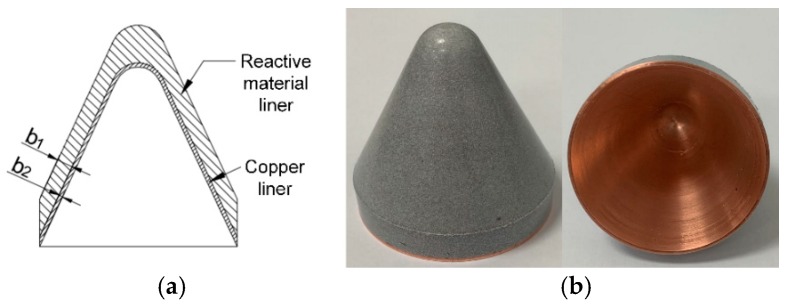
Structure and photograph of the RM-CL specimen. (**a**) Structure of liner; (**b**) Liner specimen.

The preparation process of the RM-CL mainly consisted of the following four steps: (1) the first step was to machine designed the copper liner. (2) Second, the machined copper liner was placed into the inner layer of the pre-pressed reactive material liner, and then the double-layered liner was pressed at 300 MPa for 10 min to ensure that they fit closely. (3) The pressed liner sample was relaxed at ambient pressure and temperature for not less than 24 h to remove the residual stress, and then the specimen was sintered at a temperature of 380 °C in a vacuum oven. (4) Lastly, the sintered liner specimen was reshaped to prevent the deformation of the liner during the sintering process from affecting the jet-formation performance. For the specimen, the reactive liner materials were a mixture of 73.5 wt.% PTFE and 26.5 wt.% Al powders by mass-matched ratios, and the density of the reactive liner was approximately 2.3 g/cm^3^. The average sizes of the PTFE and Al particles were approximately 28 μm and 9 μm, respectively. The reactive material liner and copper liner had the same shape, with base diameters of 50 mm and apex angles of 55°. The thicknesses of the reactive material liners (*b*_1_) were 3, 4, and 5 mm. The thickness of the copper liner (*b*_2_) was 1 mm. A typical liner specimen is shown in Figure 3.

### 3.2. Experimental Setup

**Figure 4:**
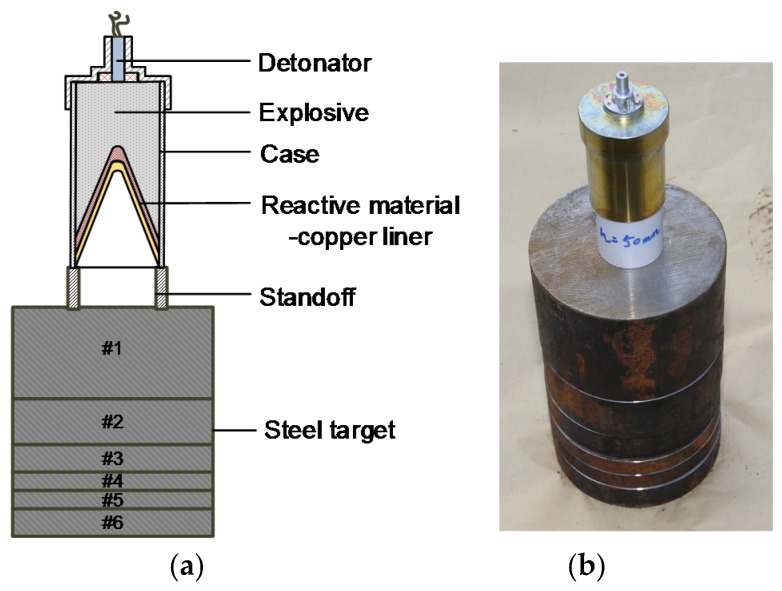
Schematic and photograph of the experimental setup. (**a**) Experimental schematic; (**b**) Experimental setup.

To investigate the penetration enhancement behaviors of the RM-CL shaped charge against steel target, experiments were conducted according to the schematic diagram shown in Figure 4. The complete warhead consisted of an outer reactive liner, an inner copper liner, explosive, a case, and a detonator. The height of the main charge was 100 mm and the explosive was initiated by a detonator placed on the center of the main charge. The case, which was machined by #45 steel, was 3 mm thick. The target was made of multi-layered steel plates with a total thickness of 250 mm; i.e., 100 + 50 + 30 + 20 + 20 + 30 mm. In order to determine the influence of reactive liner thickness on the penetration performance of RM-CL shaped charge, a series of experiments were carried out at standoffs of 1.0, 2.0, and 2.5 CD, respectively.

### 3.3. Experimental Results

The experimental results of the RM-CL shaped charges against steel targets are summarized in Table 1, in which *H*, *P*_d_, and *D*_0_ refer to the standoff, penetration depth, and hole-diameter, respectively. Table 1 indicates that the largest penetration depth is approximately 3.72 CD when the reactive liner thickness is 5 mm at a standoff of 2.5 CD. When *b*_1_ is 3 mm and 4 mm, the maximum *P*_d_ is approximately 2.90 CD and 3.40 CD, which correspond to standoffs of 2.0 CD and 2.5 CD, respectively. Accordingly, for a given copper liner thickness, with increasing reactive liner thickness, the maximum *P*_d_ of the reactive material-copper jet (RM-CJ) improves and the corresponding optimum standoff increases slightly. However, for the traditional single reactive liner shaped charge jet in the reference [24], the penetration depths of this reactive jet at the standoff of 1.0 CD, 1.5 CD, and 2.0 CD were 1.18 CD, 1.22 CD, and 0.95 CD, respectively. Thus, these experimental results indicate that, compared with the single reactive jet [24], the penetration depths of RM-CJ significantly increase. 

In addition, it also can be seen that, for the same standoff, the penetration depth of the RM-CJ grows remarkably with the increase in reactive liner thickness. Particularly, compared with the aluminum-copper jet penetrating steel target, this is an unusual experimental phenomenon. This is because, for a given 2-mm double-layered liner, the penetration capability of the aluminum-copper jet decreases with increasing thickness of the aluminum liner when the thickness exceeds 1 mm [32]. 

According to Table 1, the reactive liner thickness and standoff have a negligible effect on the entrance hole-diameter of the #1 steel plate. The average entrance hole-diameters of #1 steel plates are approximately 0.53 CD for the RM-CJs, which are smaller than those of the single reactive jets (the average entrance hole-diameters were 0.72 CD, 0.67 CD, and 0.58 CD for the standoff of 1.0 CD, 1.5 CD, and 2.0 CD, respectively) [24], whereas they are larger than those of the single copper jets (the average entrance hole-diameters are approximately 0.4 CD) [32,33]. Nevertheless, when the standoff is 1.0 CD, the entrance hole-diameters of #2 and #3 steel plates are superior to those for other standoffs. In particular, when the reactive liner thickness is 5 mm, its entrance aperture area for #2 steel plate increases by approximately 60% compared with that of 3-mm reactive liner. 

**Figure 5:**
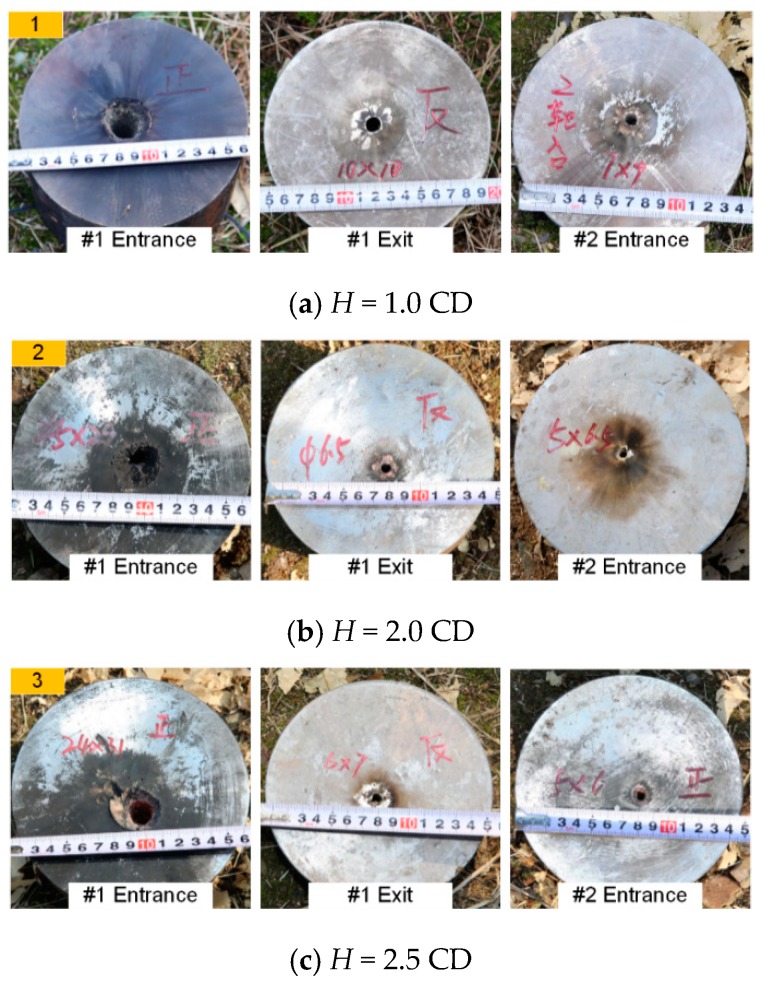
Typical experiments of RM-CL shaped charge with a 3-mm reactive liner.

**Figure 6:**
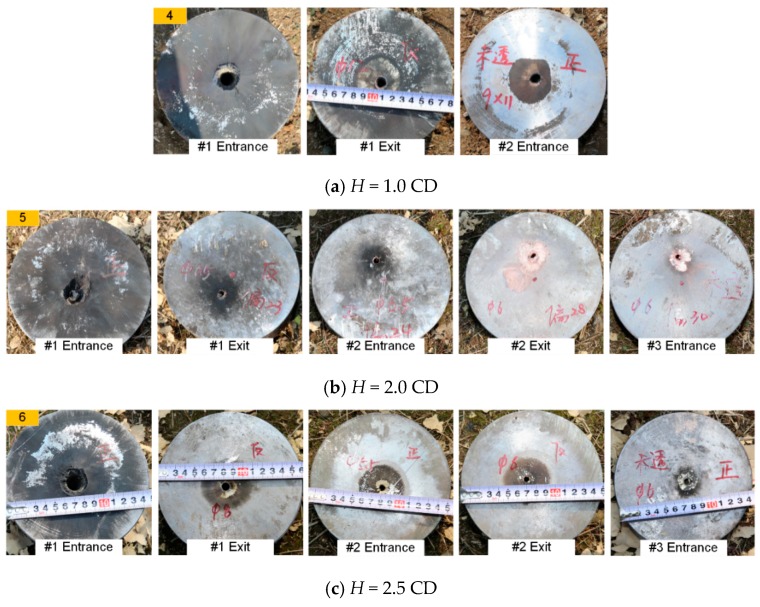
Typical experiments of RM-CL shaped charge with a 4-mm reactive liner.

**Figure 7:**
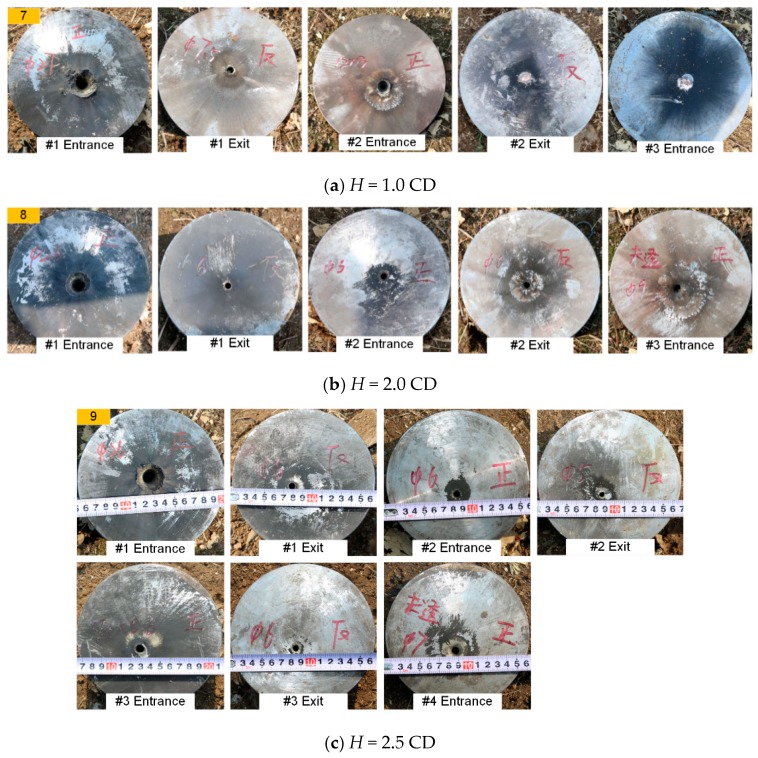
Typical experiments of RM-CL shaped charge with a 5-mm reactive liner.

Figure 5, Figure 6 and Figure 7 show typical experimental pictures of the front and back surfaces of steel plates impacted by the RM-CJ. There are black detonation products of reactive materials (namely, the black marks on the steel plates) on the front and back surfaces of each penetrated steel plate. In particular, the front surface of #1 steel plate is almost completely covered by black detonation products, and there are some small cracks at the entrance hole of #1 steel plate. In fact, the extent of the black marks on the target surfaces is proportional to the reactive material mass entering the penetrated target, and the reactive-material mass will significantly influence the damage mode of steel plates or the behind-armor damage effects based on the references of [22] and [24]. Moreover, when *b*_1_ is 3 mm, the back surface of #1 steel plate and the front surface of #2 steel plate are covered by some black marks at the standoff of 1.0 CD (see Figure 5a), while the black marks become increasingly less obvious at the standoff of 2.0 and 2.5 CD (see Figure 5b,c). When *b*_1_ is 4 mm, some black detonation product on the back surface of #1 steel plate and the front surface of #2 steel plate was present at a standoff of 1.0 CD and 2.0 CD. When the standoff is 2.0 CD, there are almost no black marks on the back surface of #2 steel plate or the front surface of #3 steel plate (see Figure 6b right), which may be owing to the poor symmetry of the double-layered liner affecting the follow-thru capability of reactive materials. However, when the standoff is 2.5 CD, only a small amount of black detonation product is observed on the back surface of #1 steel plate (see Figure 6c). In addition, when *b*_1_ is 5 mm, the black detonation product on the front surfaces of #3 steel plates are still obvious. However, compared with other standoffs, the black marks on the front surface of #3 steel plate is most obvious at a standoff of 1.0 CD (see Figure 7a).

These experimental phenomena have verified that the deflagration reaction of reactive materials may not occur during the process of jet formation, and the reactive materials can follow the precursor copper jet into the penetrated target, releasing its chemical energy inside the target during or after the termination of the penetration process. The penetration depth and reactive material mass entering the penetrated target strongly depend upon the reactive liner thickness and standoff. It is clear that the RM-CL shaped charge presents a novel defeat mechanism that incorporates the penetration capability of precursor copper jet and chemical energy release of the follow-thru reactive material penetrator, which eventually could enhance the penetration and open-cratering performance of the reactive material shaped charge. 

## 4. Penetration Enhancement Mechanism 

### 4.1. Numerical Method and Material Model

**Figure 8:**
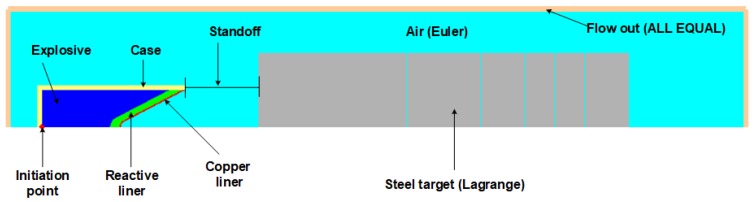
Penetration schematic of RM-CL shaped charge against steel target.

**Figure 9:**
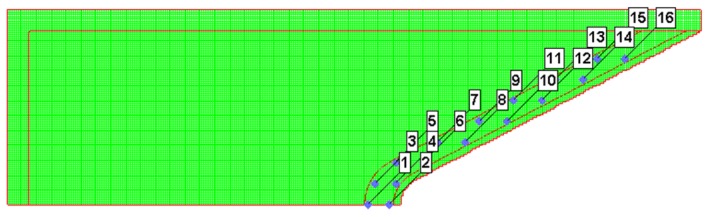
Gauge point location for the reactive liner.

To analyze the influence of the reactive liner thickness on jet formation and penetration performance of the RM-CL shaped charge an against steel target under different standoffs, a Lagrange-Eulerian model was developed based on the platform of the AUTODYN-2D code (Figure 8). The explosive, case, reactive liner, and copper liner were meshed using the Eulerian algorithm to reduce large deformation, while the target was meshed using the Lagrangian algorithm for fracture and fragmentation. The mesh used a smaller size of 0.5 mm × 0.5 mm per cell for the 50 mm × 500 mm Euler domain, and the mesh size of the steel target was 1.0 mm × 1.0 mm. The boundary condition of the air (Euler) domain was set as “Flow out (ALL EQUAL)” to eliminate the boundary effect. As shown in Figure 9, several moving gauges were set along with the generatrix on the inner and outer walls of the reactive liner to record the history of temperature. Detailed material strength models and equation of states (EOSs) of each part of RM-CL shaped charge are shown in Table 2.

The reactive liner materials were modeled using a shock equation of state. The relationship between the velocity (*U*_s_) and the particle velocity (*u*_p_) can be approximated by [34], as follows:

*U*_s_ = *c*_0_ + *Su*_p_ (3)

where *c*_0_ and *S* are based on data from plate-on-plate impact tests performed on the material. The Grüneisen parameter, *Γ*, was treated as a constant. 

The reactive liner materials were described by the Johnson–Cook strength model [34], which expresses the behavior of materials subjected to high strains, high strain rates, and high temperatures. This material model can be expressed as follows:

(4) $$\sigma_{y}=\left[A+B{\left({\bar{\varepsilon }}^{P}\right)}^{n}\right]\left[1+C\ln({\dot{\varepsilon }}^{*}\right)]\left[1-{\left(\frac{T-T_{room}}{T_{m}-T_{room}}\right)}^{m}\right]$$

where *A*, *B*, *C*, *M*, and *N* are material constants, ${\bar{\varepsilon }}^{P}$ is the effective plastic strain, ${\dot{\varepsilon }}^{*}$ is the dimensionless strain rate, *T*_m_ is the melting temperature of the material, and *T* and *T*_room_ are the ambient temperature and room temperature, respectively.

The materials of the copper and #45 steel were also used for the shock equation of state incorporating the Johnson–Cook strength model. The main material parameters of reactive liner, copper, and #45 steel are shown in Table 3. The main charge was the 8701 explosive with a nominal density of 1.71 g/cm^3^ and a detonation velocity of 8315 m/s [35], which was modeled using the Jones–Wilkins–Lee (JWL) EOS, as shown in Table 4. The 8701 explosive mainly consisted of RDX, PVAC, DNT, and CS. The material parameters of air were derived from and shown in Table 5.

### 4.2. Jet Formation Characteristics of RM-CL Shaped Charge

**Figure 10:**
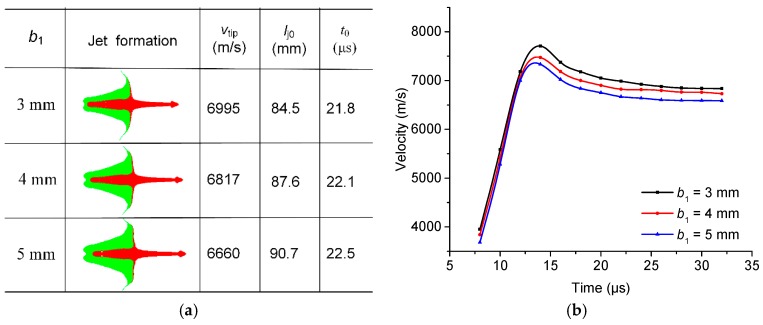
For different reactive liner thicknesses: (**a**) RM-CJ formation characteristics at standoff of 1.0 CD; (**b**) RM-CJ tip-velocity to time curves.

Figure 10a shows the RM-CJ formation characteristics of different reactive liner thicknesses before impacting the target when the standoff is 1.0 CD, where *v*_tip_, *l*_j0_, and *t*_0_ denote the jet tip-velocity, jet length, and jet formation time, respectively. The numerical results demonstrate that, for different reactive liner thicknesses, the high-velocity precursor jets are formed by the inner copper liner, and the major parts of the slugs are developed with the outer reactive material liners. With an increase of reactive liner thickness, the mass of slug increases significantly, which causes the length of RM-CJ to increase. As shown in Figure 10b, the RM-CJ tip-velocity decreases dramatically when increasing the reactive liner thickness. This is mainly because, for the same configuration of the RM-CL shaped charge, the thicker the reactive liner, the larger the mass of the double-layered liner is, and the less the mass of explosive, which results in a reduction of the jet-tip velocity to some extent.

**Figure 11:**
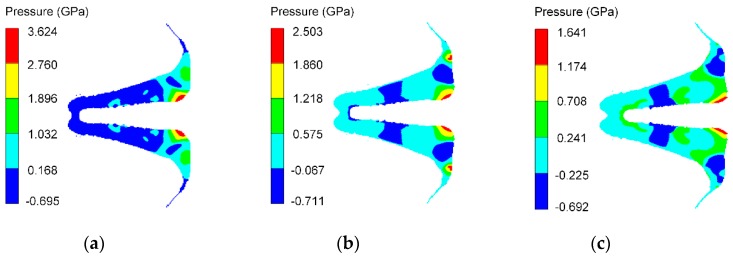
Pressure distribution of different reactive liner thicknesses at *t*_0_. (**a**) *b*_1_ = 3 mm; (**b**) *b*_1_ = 4 mm; (**c**) *b*_1_ = 5 mm.

**Figure 12:**
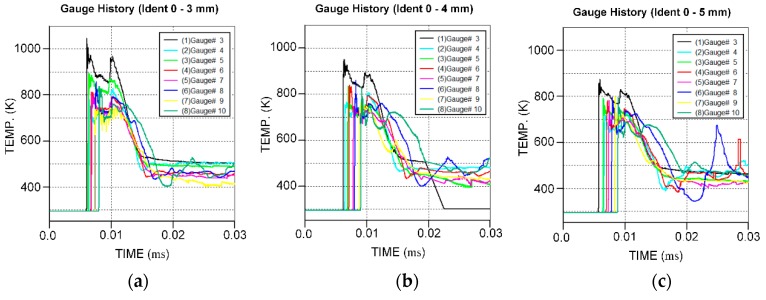
Temperature–time curves of gauges for different reactive liner thicknesses. (**a**) *b*_1_ = 3 mm; (**b**) *b*_1_ = 4 mm; (**c**) *b*_1_ = 5 mm.

Figure 11 shows the pressure distribution on the reactive materials in Figure 10a. The high-pressure areas all occur at the interfaces between the slugs of the reactive material and the copper, and the high-pressure area and the peak pressure value decrease with increasing the reactive liner thickness. Figure 12 presents the typical history temperature for gauges #3 to #12 under different reactive liner thicknesses. The numerical simulations represent that when the time increases from 5 μs to 12 μs, the temperature on the reactive material liner drops obviously with increasing reactive liner thickness, and the maximum temperature values of gauge #3 for 3-mm, 4-mm, and 5-mm reactive liner are 1038 K, 950 K, and 878 K, respectively. According to the reference [3], the PTFE will decompose starting at 400 °C with its peak at 562 °C; then, as the temperature continues to rise, the Al particles will participate in the redox reaction with the decomposition product of PTFE. Therefore, the temperature results demonstrate that, under the detonation shock wave of explosive, the PTFE/Al reactive materials were activated in the jet formation process. 

### 4.3. Comparison of Simulated Results and Experimental Penetration Depth

**Figure 13:**
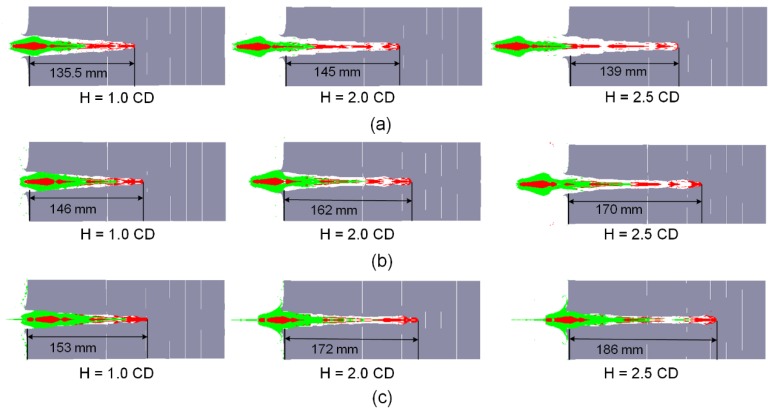
Numerical simulation results of the RM-CJ penetrating steel target. (**a**) *b*_1_ = 3 mm; (**b**) *b*_1_ = 4 mm; (**c**) *b*_1_ = 5 mm.

To further analyze the penetration behaviors of the RM-CL shaped charge against a steel target, the penetration results of the above experimental conditions were simulated using the AUTODYN-2D code. When the numerical penetration depths fit well with the experimental results, the simulated results of RM-CJ inside the penetrated steel target are illustrated in Figure 13. Figure 13 shows that, for different reactive liner thicknesses and standoffs, it is always the high-velocity copper jets that penetrate the steel targets, whereas the reactive jet penetrators can follow the copper jets into the penetration holes but do not begin impacting the targets. Therefore, under the experimental conditions, the penetration termination of the RM-CL shaped charge against steel target is that the reactive materials undergo the deflagration reaction during the penetration process and produce large amounts of gas and chemical energy, resulting in a serious instability of the precursor copper jet, which eventually leads to the residual jet not being able to continue penetration; this leads to an early termination of the penetration process. 

**Figure 14:**
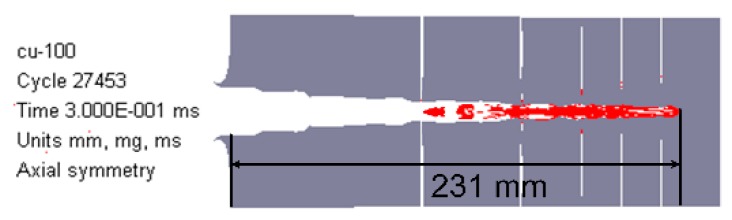
Penetration performance of the single copper jet (*b*_1_ = 0) at standoff of 1.0 CD.

Furthermore, it is obvious from Figure 13, that if the deflagration reaction of reactive materials is not considered, the precursor copper jets would continue to penetrate the steel targets. By means of the numerical simulation, if the thickness of reactive liner is zero (namely *b*_1_ = 0). The tip-velocity of the single copper jet is approximately 7580 m/s, which is larger than that of the RM-CJ, and this will produce the deeper penetration depth, as shown in Figure 14. However, for the RM-CL shaped charge technology, its damage mechanism is different from the penetration by only kinetic energy of the copper jet, and it is also different from the single reactive jet [24] that incorporates the kinetic energy penetration of reactive jet and the chemical energy of reactive materials. The enhanced damage mechanism of the RM-CL shaped charge combines the penetration capability of a precursor copper jet and the chemical energy release of a follow-thru reactive material penetrator. Thus, compared with the single reactive jet, the penetration enhancement of the RM-CJ strongly depends on the penetration capability of the precursor jet, owing to the copper jet with high density and excellent ductility. Although the penetration hole-diameter of RM-CJ is smaller than that of the single reactive jet [24], the follow-thru reactive material penetrator will still produce extremely high pressure inside of the penetrated target, into which the reactive materials will release the heat to the fullest, resulting in efficient damage to various desired targets.

Figure 13 also shows that, for a given thickness of reactive liner, when the standoff increases from 1.0 CD to 2.5 CD, the mass of the reactive materials entering the penetration hole will dramatically decrease. Especially, when the reactive liner thickness is 3 mm, all reactive materials are outside the target at the standoff of 2.5 CD, as seen in Figure 13a (H = 2.5 CD), which cannot fully exert the advantage of deflagration performance for the RM-CL shaped charge. Moreover, for the same standoff, the mass of reactive materials entering the penetration hole increases significantly when increasing the reactive liner thickness. However, compared with the 3-mm reactive liner, when the reactive liner thickness is 4 and 5 mm, a few of reactive materials are blocked outside the penetration hole, resulting in a reduction in the utilization rate of reactive liner (see Figure 13c). For the RM-CL shaped charge technology, its excellent damage effects not only need to ensure that the jet can perforate the steel casing of the target, but also allow more reactive materials to enter the penetrated target, which will achieve greater devastating damage to the target. Therefore, the thicker the reactive liner thickness, the larger the corresponding optimum standoff of RM-CJ is, which can fully exploit the combined defeat mechanisms of kinetic energy and chemical energy of the RM-CL shaped charge to ensure sufficient penetration capability, and more reactive materials undergoing deflagration inside of the target.

In addition, according to the experimental results, the average entrance hole-diameter of the #1 steel plate is approximately 0.53 CD for the RM-CJ, which is approximately 51.4% larger than that of the single copper jet with a cone angle of 55° (0.35 CD) [33]. It is approximately 35.9% and 20.5% larger than that of the single copper jet and aluminum-copper jet with a cone angle of 60° (0.39 CD and 0.44 CD, respectively) [32]. Simultaneously, compared with the simulated average hole-diameter of 0.46 CD (see Figure 13), the experimental results increase by approximately 15.2%. These data indicate that, for the same cone angle of the liner, compared with the copper jet and the aluminum-copper jet, the penetration hole-diameters of RM-CJ increase significantly, and the experiments also demonstrates that the deflagration of reactive materials will produce secondary expansion cratering effects. In particular, when the reactive liner thickness is 5 mm at a standoff of 1.0 CD, the re-expansion cratering effects will obviously enhance. This is mainly because for the thicker reactive liner at a lower standoff, there is more reactive-materials mass entering the penetration hole (see Figure 13, *H* = 1.0 CD), which leads to the higher pressure produced by reactive materials deflagrating inside the initiated penetration hole.

Based on the above discussions, the enhanced damage effects of an RM-CL shaped charge against steel target mainly embody in the following three aspects: (1) Compared with the traditional single reactive jet, the penetration depth of RM-CJ increases significantly; (2) compared with the copper jet or the aluminum-copper jet, the penetration hole-diameter of RM-CJ greatly improves; and (3) the experimental results show that the reactive materials can enter the penetrated steel plates. Therefore, it is reasonable to infer that if the RM-CJ can perforate the steel plate with limited thickness, the reactive materials entering inside the target will produce greater damage effects.

### 4.4. Initiation Delay Time Effects on Penetration Performance

**Figure 15:**
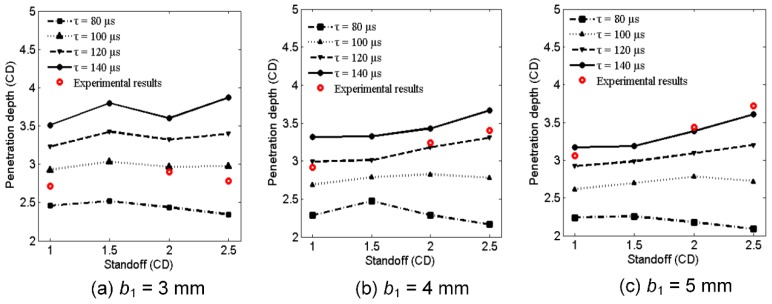
Influence of reactive liner thickness on initiation delay time and penetration depth.

According to numerical simulations, the penetration depths of different initiation delay times (*τ*) are shown in Figure 15. For a given standoff, if the initiation delay time is long enough, the penetration depth of the RM-CL shaped charge with a 3-mm reactive liner is always larger than that of the 4-mm and 5-mm reactive liners, owing to the thinner reactive liner with a higher jet tip-velocity (see Figure 10b). This is consistent with the penetration mechanism of the aluminum-copper jet against a steel target [32]. However, according to the above experimental results, the penetration depth of the RM-CL shaped charge increases with an increase in the reactive liner thickness. As such, for the RM-CL shaped charge technology, compared with the jet’s characteristics, the initiation delay time of reactive materials has a much greater influence on its penetration performance. 

When the penetration depths of the simulations are fit with the experimental results (see the red circles in Figure 15), the initiation delay time can be calculated by averaging the three simulated penetration times, and the initiation delay times of 3-mm, 4-mm, and 5-mm reactive liners are approximately 94.7, 120.4, and 140.5 μs, respectively. The simulated and experimental results indicate that the reactive liner thickness has a significant influence on the initiation delay time of reactive materials, increasing markedly with increasing the reactive liner thickness. The key reason for the result is that the thicker reactive liner will be subjected to a lower temperature during the jet formation process (see Figure 12), eventually decreasing the decomposition and redox reaction rate of the reactive materials, and resulting in an increase in the initiation delay time [5,7]. Another important consideration is that, due to the smaller jet tip-velocity of the thicker reactive liner, the reactive materials are subjected to a lower secondary impact pressure when the jet impacts the target (see Figure 11), which will again decrease the chemical reaction rate of the reactive materials. Hence, the lower temperature or pressure inside the reactive materials is likely to be an important mechanism for increasing the initiation delay time, which will eventually enhance the penetration damage effects of the RM-CL shaped charge. It should be noted, however, that under the action of strong shock wave generated by the 8701 explosive detonation, it is assumed in this paper that the reactive material penetrator will be inert during the jet formation and penetration process, and all the reactive materials would deflagrate simultaneously and release their chemical energy instantaneously at the time of *τ*, ignoring the propagation of deflagration reaction rate within the reactive material penetrator described in reference [36].

**Figure 16:**
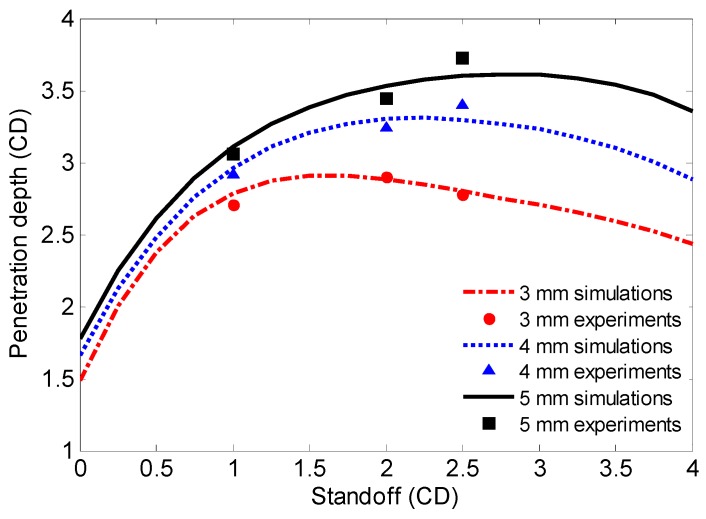
Initiation delay time affecting on penetration depth and optimum standoff.

To further analyze the influence of the initiation delay time on the penetration performance and optimum standoff of RM-CJ, the curves between the penetration depth and standoff for different reactive liner thicknesses are shown in Figure 16. As the standoff increased from 0 to 4.0 CD, the penetration depths were simulated every 0.25 CD based on the above calculated initiation delay time, and three curves can be obtained by fitting these calculated values using the fourth-order polynomial. Figure 16 demonstrates that, for a given thickness of copper liner, if considering only the penetration depth, the optimum standoff of RM-CL shaped charge increases with increasing the reactive liner thickness. That is to say, the initiation delay time significantly influences the optimum standoff of RM-CJ. Figure 16 also illustrates that, for the same standoff and a given thickness of copper liner, when the reactive liner thickness increases from 3 mm to 5 mm, the penetration depth of RM-CJ increases significantly, but the rate of increase gradually decreases. This is mainly because a longer initiation delay time can be obtained using a thicker reactive liner (see Figure 15), whereas this method could dramatically decrease the tip-velocity of the RM-CJ (see Figure 10b). However, based on the penetration theory of reactive jet [24], the penetration depth is proportional to not only the jet-tip velocity but also the initiation delay time of reactive materials. Hence, for this RM-CL shaped charge technology, a reasonable thickness ratio of the reactive liner to the copper liner is important to ensure sufficient jet-tip velocity and relatively longer initiation delay time, resulting in deeper penetration depth and more reactive materials entering the penetrated target, eventually enhancing the penetration damage effects.

In addition, it can be seen from Figure 16 that the experimental results generally agree well with the numerical simulations, but the experimental penetration depth results are all smaller than those of the simulations when the standoff is 1.0 CD. Excluding the experimental errors, the main reason is that, when the standoff is smaller, it will take less time to form a jet and the jet tip-velocity is relatively larger, which leads the jet to impact the target early. Moreover, the reactive materials will be subjected to a higher secondary impact pressure, accelerating the deflagration reaction of reactive materials and declining the initiation delay time. Consequently, when the standoff is 1.0 CD, the actual initiation delay times of the experiments are less than the calculated average penetration time, resulting in the experimental results all being smaller than the simulated penetration depths. 

According to the above discussions, for the double-layered liner shaped charge with the reactive material liner, prolonging the initiation delay time of reactive materials is important. However, the initiation delay time is influenced not only by the formulation and the particle size of reactive materials, but also by the structure of this shaped charge and the action condition of the jet impacting the target. Therefore, when a novel reactive material liner shaped charge will be designed, the initiation delay time of reactive materials should be actively increased, which can make full use of the combined damage effects of the chemical and kinetic energy to achieve enhanced damage to the thick targets. 

## 5. Conclusions

The penetration enhancement behaviors of reactive material-copper liner shaped charge against a steel target were studied, and its penetration performance and damage mechanism were investigated by both experiments and simulations. Several conclusions are presented as follows:

(a) Experimental results have shown that the penetration depth of a reactive material-copper jet increased significantly compared with the single reactive jet, and the penetration hole-diameter was larger than that of the single copper jet; and the reactive materials can enter the penetrated target. These phenomena have verified that this RM-CL shaped charge can incorporate the kinetic energy of a copper jet and the chemical energy of follow-thru reactive materials to achieve a deeper penetration depth and damage enhancement effects.

(b) For the same standoff and a given copper liner thickness, the penetration performance of a reactive material-copper jet and the mass of reactive materials entering the penetrated target positively related to the reactive liner thickness. It indicated that the reasonable thickness ratio of the reactive liner to the copper liner is important to ensure that the jet can perforate the limited thickness target and more reactive materials can enter the penetrated target, enhancing the penetration damage effects of the RM-CL shaped charge.

(c) For the RM-CL shaped charge technology, its penetration enhancement behavior is strongly dependent upon the initiation delay time of reactive materials. For mechanism considerations, with increasing the reactive liner thickness, the reactive liner would be subjected to a lower temperature and secondary impact pressure, which decreased the rate of deflagration reaction and led to the initiation delay time being prolonged, resulting in an increase in the penetration depth.

## Figures and Tables

**Table 1:** Experimental results of RM-CJ penetrating steel targets.

No.	*b*_1_ (mm)	*H* (CD)	*P*_d_ (CD)	*D*_0_ (mm)						
				#1		#2		#3		#4
				Entrance	Exit	Entrance	Exit	Entrance	Exit	Entrance
1	3	1.0	2.71	Φ25.7	Φ10	7 × 9	-	-	-	-
2	3	2.0	2.90	25 × 29	Φ6.5	5 × 6.5	-	-	-	-
3	3	2.5	2.78	24 × 31	6 × 7	5 × 6	-	-	-	-
4	4	1.0	2.92	Φ26	Φ12	9 × 11	-	-	-	-
5	4	2.0	3.24	Φ25	Φ6.5	Φ6.5	Φ6	Φ6	-	-
6	4	2.5	3.40	Φ25	Φ8	Φ5.5	Φ6	Φ6	-	-
7	5	1.0	3.06	Φ27	Φ7.5	12 × 13	Φ12	Φ14	-	-
8	5	2.0	3.44	Φ26	Φ6	Φ6	Φ6	Φ9	-	-
9	5	2.5	3.72	Φ26	Φ6	Φ6	Φ5	7.5 × 8.6	Φ6	Φ7

**Table 2:** Material strength models and EOSs of each part of RM-CL shaped charge.

Part	Materials	EOS	Strength Model	Erosion
Air	Air	Ideal Gas	None	None
Outer liner	Reactive materials	Shock	Johnson Cook	None
Inner liner	Copper	Shock	Johnson Cook	None
Explosive	8701	JWL	None	None
Case	#45 steel	Shock	Johnson Cook	None
Steel target	#45 steel	Shock	Johnson Cook	Geometric Strain 1.5

**Table 3:** Parameters of the reactive liner [24,34], copper [35], and #45 steel [24] materials.

Materials	*ρ* (kg/m^3^)	*G* (GPa)	*A* (MPa)	*B* (MPa)	*n*	*C*	*m*	*T*_m_ (K)	*T*_room_ (K)	Γ	c_0_ (m/s)	S
Reactive liner	2.27	0.67	8.04	250.6	1.8	0.4	1	500	294	0.9	1450	2.2584
Copper	8.97	46.5	90	292	0.31	0.025	1.09	1356	293	2.02	3940	1.49
#45 steel	7.83	77	792	510	0.26	0.014	1.03	1793	300	2.17	4570	1.49

**Table 4:** Parameters of the 8701 explosive [24,35].

Material	*ρ* (kg/m^3^)	*D* (km/s)	*P*_CJ_ (GPa)	*E* (GPa)	*A* (GPa)	*B* (GPa)	*R*_1_	*R*_2_	*ω*	*v*_0_
Explosive	1.71	8.315	28.6	8.499	524.23	7.678	4.2	1.1	0.34	1.00

**Table 5:** Material parameters of air [24].

Material	*ρ* (kg/m^3^)	*γ*	*C*_p_ (kJ/kg·K)	*C*_v_ (kJ/kg·K)	*T* (K)	*E*_0_ (kJ/kg^−1^)
Air	1.225	1.4	1.005	0.718	288.2	2.068 × 10^5^
